# RhoH Regulates Subcellular Localization of ZAP-70 and Lck in T Cell Receptor Signaling

**DOI:** 10.1371/journal.pone.0013970

**Published:** 2010-11-12

**Authors:** Hee-Don Chae, Jamie E. Siefring, David A. Hildeman, Yi Gu, David A. Williams

**Affiliations:** 1 Division of Experimental Hematology, Cincinnati Children's Research Foundation, Cincinnati Children's Hospital Medical Center and University of Cincinnati College of Medicine, Cincinnati, Ohio, United States of America; 2 Division of Immunobiology, Cincinnati Children's Research Foundation, Cincinnati Children's Hospital Medical Center and University of Cincinnati College of Medicine, Cincinnati, Ohio, United States of America; New York University, United States of America

## Abstract

RhoH is an hematopoietic-specific, GTPase-deficient Rho GTPase that plays a role in T development. We investigated the mechanisms of RhoH function in TCR signaling. We found that the association between Lck and CD3ζ was impaired in RhoH-deficient T cells, due to defective translocation of both Lck and ZAP-70 to the immunological synapse. RhoH with Lck and ZAP-70 localizes in the detergent-soluble membrane fraction where the complex is associated with CD3ζ phosphorylation. To determine if impaired translocation of ZAP-70 was a major determinant of defective T cell development, *Rhoh^-/-^* bone marrow cells were transduced with a chimeric myristoylation-tagged ZAP-70. Myr-ZAP-70 transduced cells partially reversed the *in vivo* defects of RhoH-associated thymic development and TCR signaling. Together, our results suggest that RhoH regulates TCR signaling via recruitment of ZAP-70 and Lck to CD3ζ in the immunological synapse. Thus, we define a new function for a RhoH GTPase as an adaptor molecule in TCR signaling pathway.

## Introduction

RhoH, an hematopoietic-specific Rho GTPase,was first identified as a fusion transcript with Bcl-6 in B-cell diffuse large cell lymphoma[1]. RhoH is a member of RhoE/Rnd3 subfamily that has no intrinsic GTPase activity and remains in a GTP-bound and constitutively active state. RhoH cellular function has been reported to be controlled at both the transcriptional and post-translational levels[2], [3], [4]. For example, RhoH mRNA is down-regulated after phorbol myristate acetate treatment in Jurkat T cells and after stimulation of T cell receptor (TCR) in Th1 cells[4].

RhoH has also been proposed to function as a negative regulator of other Rho GTPases, particularly Rac. For instance, RhoH is a potent inhibitor of the activation of NF*_κ_*B and p38 kinase by other Rho GTPases in Jurkat T cells[4]. Overexpression of RhoH impairs the activation of Rac GTPases and negatively regulates the Rac-mediated cortical F-actin assembly and chemotaxis in hematopoietic progenitor cells (HPCs)[5], [6]. The level of activated Rac GTPases is elevated in *Rhoh* ^-/-^ HPCs[5].

We previously demonstrated that *Rhoh* ^-/-^ mice have impaired TCR signaling, resulting in defective thymocyte selection and blocked maturation of T cells[3]. CD3ζ phosphorylation and translocation of ZAP-70 to the immunological synapse are impaired in RhoH-deficient T cells, which results in the defective activation of components of ZAP-70-mediated signaling pathway including Linker for Activation of T cells (LAT) and p44/42 MAP kinase[3], [7]. Upon TCR stimulation, RhoH is phosphorylated on tyrosine residues within an immunoreceptor tyrosine-based activation motif (ITAM)-like motif which leads to enhanced interaction of RhoH and ZAP-70[3]. Thus, RhoH plays a critical role in TCR signaling and activation of ZAP-70 and is essential for T cell development and TCR-mediated function.

TCR signaling is controlled by complex cross-regulation of several key downstream signaling components. ZAP-70 has been reported to promote the phosphorylation of CD3ζ and the association of CD3ζ with Lck, as ZAP-70 deficiency impairs the TCR-induced CD3ζ phosphorylation[8], [9], [10]. Although RhoH-deficient T cells also have defective recruitment of ZAP-70 to TCR and CD3ζ phosphorylation is impaired in *Rhoh* ^-/-^ T cells[3], the molecular underpinnings of how RhoH regulates Lck-mediated CD3ζ phosphorylation via ZAP-70 in TCR signaling remain unclear. Here, we investigated further the role of RhoH in localizing ZAP-70 to the TCR and the potential role of RhoH in Lck-mediated CD3ζ activation. In *Rhoh* ^-/-^ T cells, Lck showed impaired association with CD3ζ and decreased localization to the immunologic synapse. This was not associated with defects in its kinase activity as measured by in vitro kinase assay. Further biochemical data suggest that RhoH regulates CD3ζ phosphorylation by recruiting ZAP-70 and Lck to the cell membrance fraction to form the TCR complex for TCR signaling activation. In these studies, we found that membrane-targeted ZAP-70 could partially rescue the defective TCR signaling in RhoH-deficient T cells. Taken together, these data suggest that RhoH regulates TCR signaling via recruiting ZAP-70 and Lck to CD3ζ in the immunological synapse for activation of downstream signaling molecules.

## Materials and Methods

### Mice and T lymphocyte isolation

129Sv *Rhoh^-/-^* mice were generated by standard DNA homologous recombination and backcrossed in a C57BL/6J background as described previously[3]. p14^tg/+^; *Rhoh* ^-/-^ mice expressing p14 TCR (Vα2/Vβ8) transgene in a compound 129Sv and C57BL/6J background were generated as described previously[3]. Animals used were littermates derived from N20 backcross generation. 129S6/SvEvTac-*Rag2* ^-/-^ mice were purchased from Taconic Animal Models (Hudson, NY). All experiments involving animals were approved by the Institutional Animal Care and Use Committee of the Cincinnati Children's Research Foundation. Single cell suspensions were generated from dissected thymus and lymph node. T cells were purified by negative selection using the Pan T Cell Isolation Kit (Miltenyi Biotec, Auburn, CA). The purity of isolated T cells in all experiments was greater than 90%, as assessed by flow cytometry with anti-mouse CD3ε Ab. For TCR activation, thymocytes or T cells were incubated with anti-mouse CD3ε mAb (2c11, BD Pharmingen, San Diego, CA) and anti-mouse CD28 mAb (37.51, eBioscience, San Diego, CA) (both at 5 µg/10^7^ cells). Cross-linking of mAbs was accomplished using anti-Hamster IgG Abs (5 µg/10^7^ cells, BD Pharmingen). After incubation for 2 min at 37°C, cold phosphate-buffered saline (PBS, 10 vol) was added to stop the activation.

### Retroviral vector construction and retrovirus supernatant generation

Myr-ZAP-70 was constructed by the addition of the avian src myristoylation sequence (MGSSKSKPK) at the N terminus of ZAP-70 cDNA[11] (kindly provided by Dr. Andrey Shaw, Washington University). Myr-ZAP-70 was amplified using a human full-length ZAP-70 cDNA with the following primers; (forward) 5′-GACGAATTCATGGGCAGCAGCAAGAGCAAGCCCAAGCCCGATCCCGCGGCGCAC-3′ (MGSSKSKPK coding sequence was underlined), (reverse) 5′-ACCGTCGACTCAGCCACATGCAGCCTCGGCCACCTGT-3′. Amplified PCR products were subcloned in pSF91 bicistronic retroviral vector expressing enhanced green fluorescence protein (EGFP) at EcoRI and SalI sites[12]. To produce the retroviral vector expressing wild type ZAP-70, the EcoRI/SalI fragment of full-length ZAP-70 in pGEM3Z was ligated into pSF91. The sequence and orientation of the DNA construct was verified by DNA sequencing. The MIEG3-HA-RhoH vector was described previously[6]. Recombinant retroviruses were produced using the ecotropic Phoenix packaging cell system[13]. Briefly, 8 µg retroviral vector plasmid DNA, 10 µg Moloney leukemia virus (MLV) gag-pol plasmid, and 3 µg ecotropic envelope plasmid were cotransfected into Phoenix cells using the CaPO_4_ coprecipitation procedure (Invitrogen, Carlsbad, CA). Retroviral supernatants were collected every 12 hours. Titer of recombinant retrovirus was determined by infecting NIH3T3 cells using serial dilution[14].

### Mouse bone marrow transduction and reconstitution of *RAG2^-/-^* mice

Low density bone marrow (LDBM) cells were harvested 4 days after 5-fluorouracil (5-FU, 150 mg/kg) injection. Retrovirus-mediated transduction of mouse LDBM cells was performed as described previously[15]. Briefly, LDBM cells were cultured for 2 days in Iscove's Modified Dulbecco's Medium (Invitrogen) supplemented with 10% fetal calf serum (FCS, HyClone, Logan, UT), 2% penicillin and streptomycin (P/S), and 100 ng/ml each of recombinant rat stem cell factor (SCF), megakaryocyte growth and development factor (MGDF) and granulocyte colony-stimulating factor (G-CSF) (all from Amgen, Thousand Oaks, CA). Pre-stimulated LDBM cells were infected twice with the high-titer retrovirus supernatant on fibronectin fragment CH296 (kindly provided by Takara Bio, Otsu, Japan). EGFP^+^ cells were sorted by a fluorescence-activated cell sorting (FACS) Vantage Sorter (BD Biotechnology, Franklin Lakes, NJ) and were injected intravenously into the sub-lethally irradiated (300 Rads using a ^137^Cs irradiator) *Rag2* ^-/-^ recipient mice. At 8 weeks post transplantation, single cell suspensions were prepared from thymus and subjected to flow cytometric analysis.

### Antibodies

The following antibodies were used in immunoblot analyses at 1∶1000 dilution: anti- ZAP-70 (1E7.2) and anti-phosphotyrosine (4G10) (Millipore, Billerica, MA); anti-HA (3F10) (Roche, Indianapolis, IN); anti-β-actin (AC15) (Sigma, St Louis, MO); anti-CD3ζ (3F67) and anti-Lck (3A5) (Santa Cruz Biotechnology, Santa Cruz, CA); anti-phospho-Src Tyr416 (2101), anti-phospho-ZAP-70 Tyr319 (2701), anti-phospho-ZAP-70 Tyr493 (2704), anti-Lck (2752), anti-ZAP-70 (99F2), anti-phospho-LAT Tyr191 (3584)anti-phospho-p44/p42 Thr202/Tyr204 (9101) and anti-p42/p44 (9102) (Cell Signaling Technology, Beverly, MA). Primary antibodies were detected with horseradish peroxidase-conjugated goat anti-mouse (Cell Signaling Technology), or goat anti-rabbit (Cell Signaling Technology), donkey anti-rat (Santa Cruz Biotechmology) or donkey anti-goat secondary (Santa Cruz Biotechmology) antibodies (1∶3000 dilution) with enhanced chemiluminescence detection (Cell Signaling Technology) The following fluor-conjugated monoclonal anti-mouse antibodies (mAb) were used for flow cytometry: CD4 (RM4-5), CD8a (53-6.7), and CD3ε mAb (2c11) (all from BD Pharmingen).

### T cell–APC conjugation and immunofluorescence staining

Lymph node cells were isolated from WT and *Rhoh^-/-^* p14 transgenic mice and positively selected for CD8+ T cells by a magnetic bead cell selection technique (Miltenyi Biotec, Auburn, CA). For positive selection of CD8+ T cells, lymph node T cells were labeled with biotinylated anti-CD8a mAb. These cells were then further incubated with anti-biotin magnetic beads (Miltenyi Biotec) and purified in accordance with the manufacturer's recommendations. Antigen-presenting (APC) cells (CHB.2B B cell lymphoma cell line)[16] were pre-loaded with 1 µg/ml of gp33 peptide for 12 hours. The pre-loaded CHB.2B cells were mixed with CD8+ T cells from WT and *Rhoh^-/-^* p14 transgenic mice and then layered onto poly-L-lysine-coated coverslips. After 5 min, cells were fixed with BD Cytofix/Cytoperm (BD Biosciences, San Jose, CA)) and stained with Rhodamine (TRITC)-labeled phalloidin (Molecular Probes, Eugene, OR), anti-ZAP-70 (Cell Signaling) or anti-Lck (3A5, Santa Cruz Biotechnology) followed by anti–rabbit Alexa488 or anti-mouse Alexa555 (Molecular Probes). For the localization of Lck and ZAP-70 in *Rhoh^-/-^* T cells expressing Myr-ZAP-70, pan-T cells were isolated from LN by using Pan-T Cell Isolation Kit (Miltenyi Biotec). T cells were transduced with retroviruses co-expressing EGFP and Myr-ZAP-70 or EGFP alone as described previously[3]. EGFP^+^ T cells were sorted with a FACSVantage and then were cultured without anti-CD3ε Ab stimulation (IMDM with 10% FCS, 20 ng/ml IL2 (Peprotech, Rocky Hill, NJ) and 10 ng/ml IL7 (Peprotech)) for 2 days. EGFP^+^ T cells were incubated with biotin-labeled anti-mouse CD3ε mAb (2c11, BD Pharmingen) and anti-mouse CD28 mAb (37.51, eBioscience), and then conjugated with Dynabeads Biotin Binder (1∶1 ratio, Invitrogen). T-microbeads conjugates were layered onto poly-L-lysine coated coverslips and incubated at 37°C for 5 min. Cells were fixed with BD Cytofix/Cytoperm (BD Biosciences) and stained with anti-ZAP-70 or anti-Lck Ab (Cell Signaling) followed by anti–rabbit Alexa555 (Molecular Probes). Z series fluorescence images were captured with a Leica DMIRB fluorescence microscope equipped with a 40x/0.55 NA air objective lens (Leica, Wetzlar, Germany), a Hamamatsu C4742 digital camera (Hamamatsu Photomics, Hamamatsu City, Japan) and a deconvolution system driven by Openlab software (Improvision, Lexington, MA). Deconvoluted images were processed using Adobe Photoshop 6.0 (Adobe Systems, San Jose, CA). At least 100 cells were analyzed per condition.

### Subcellular fractionation

Cells were lysed by brief sonication in 200 µl ice-cold buffer A (250 mM sucrose, 20 mM Tris, pH 7.8, 10 mM MgCl_2_, 1 mM EDTA, 1 mM Na_3_VO_4_, 10 mM NaF, and complete protease inhibitor cocktail (Roche)). After centrifugation for 5 min at 900 g, took 200 µl post-nuclei (PN) fraction. PN fraction was separated into particulate membrane-containing (P100) and cytosol (C) fractions by centrifugation for 30 min at 100,000× *g*. Take 200 µl supernatant as C fraction. Pellet was solubilized with 200 µl Mg^2+^ lysis/wash buffer (Millipore) and separated by additional 30 min at 100,000× *g*. The supernatant was kept as detergent-soluble membrane (SM) fraction. The pellet was further solubilized with 200 µl 0.05% sodium dodecyl sulfate (SDS)-containing Mg^2+^ lysis/wash buffer, and then centrifuged for 30 min at 13,000× *g.* The supernatant was kept as detergent-insoluble membrane (IS) fraction. Equal volumes of each fraction were used for immunoprecipitation and immunoblotting. For immunoprecipitation, each fraction was 5-fold diluted with SDS-containing Mg^2+^ lysis/wash buffer (final SDS concentration was 0.01%).

### Immunoprecipitation and Lck kinase assay

Wild-type and *Rhoh^-/-^* thymocytes or lymph node derived T cells were left unstimulated or stimulated anti-CD3ε and CD28 mAbs for 2 min. HEK 293 cells were transfected using the CaPO_4_ coprecipitation procedure (Invitrogen). Jurkat T cells were transfected by electroporation (300 V, 960 µF, Bio-Rad). The expression vector for kinase-dead ZAP-70 (pCDNA3-KA-ZAP-70) and constitutively active ZAP-70 (pCDNA3-AA-ZAP-70) was kindly provided by Dr. A. Weiss[17]. The pSM-CA-Lck (constituve active Lck) and pSM-KD-Lck (kinase-dead Lck) were gifted from Dr. M.J. Bijlmakers[18]. For the transfection experiments, the following plasmids were used: HA–RhoH, pCDNA3-ZAP-70, pCDNA3-KA-ZAP-70, pCDNA3-AA-ZAP-70, pSM-CA-Lck and pSM-KD-Lck. Cells were lysed in ice-cold Mg^2+^ lysis/wash buffer (Upstate Biotechnology) containing mixture of protease and phosphatase inhibitors (1 mM Na_3_VO_4_, 10 mM NaF, complete protease inhibitor cocktail). Cellular lysates (500 µg) were immunoprecipitated, using the anti-Lck (1 µl, 2752, Cell Signaling), anti-HA (100 ng, 3F10, Roche) or anti-ZAP-70 (1 µg, 1E7.2, Millipore) antibodies. The immunoprecipitated mixture or 20 µg total lysates were separated on a SDS polyacrylamide gel, and transferred to a polyvinylidene difluoride membrane (PVDF) (BioRad). The membranes were probed with antibodies indicated in each experiment. For Lck kinase assay, anti-Lck immunoprecipitate products were washed three times with lysis buffer and once with kinase buffer (50 mM Tris pH 7.5, 5 mM MgCl_2_, 5 mM MnCl_2_, 1 mM Na_3_VO_4_ and 1 mM DTT) and resuspended with 30 µL kinase buffer. Reaction was started by addition of 10 µCi [γ-^32^P] ATP and left for 10 min. Reaction mixtures were subjected to SDS-PAGE and analyzed by autoradiography.

### Statistical Analysis

Each experiment was performed two-four times. Representative data or images from one of the repeated experiments were shown in the figures. Statistical significance was determined by a Student t-Test and data with p-value less than 0.05 are considered as significant difference.

## Results

### Defective interaction between Lck and CD3ζ in RhoH-deficient T cells

**Figure 1:**
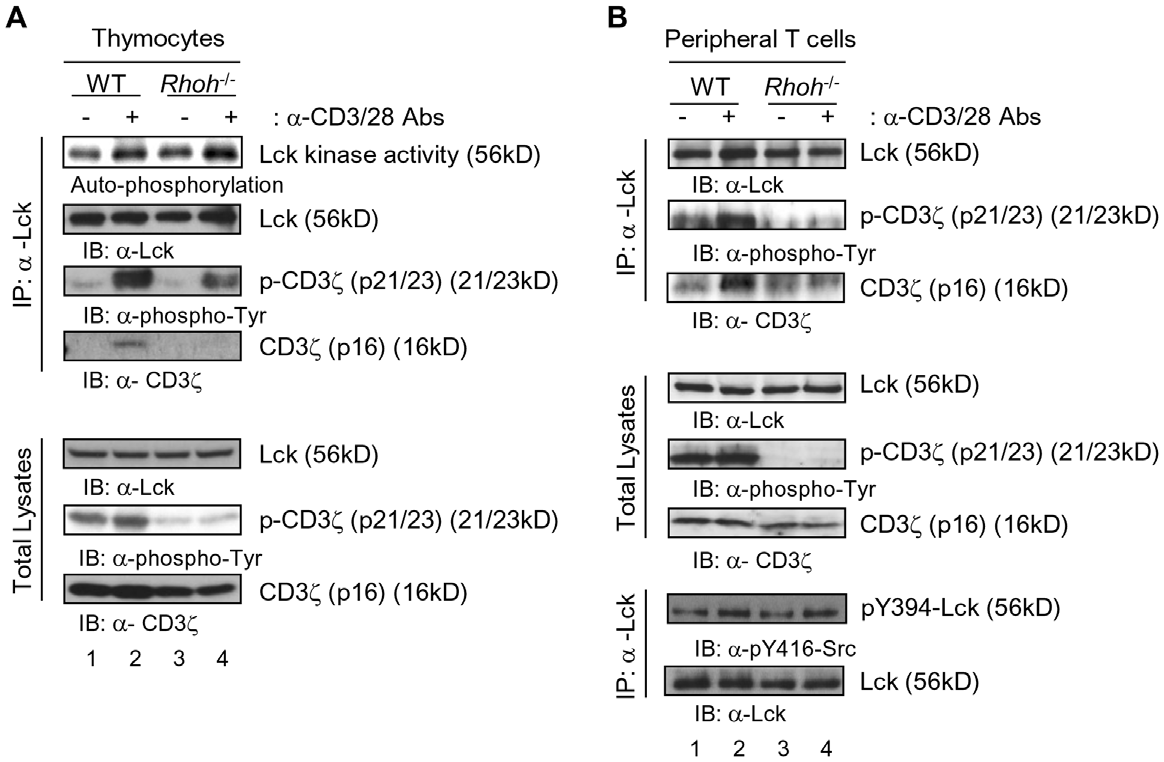
RhoH mediates the interaction of Lck and CD3ζ. Thymocytes (A) and lymph node (LN)-derived T cells (B) from wild type (WT) and *Rhoh^-/-^* mice were left unstimulated or stimulated with anti-CD3/28 Abs for 2 min. Lck autophosphorylation activity was measured by immune complex kinase assay with [γ-^32^P]ATP. Total lysates were submitted to immunoprecipitation (IP) of Lck and immunoprecipitates and total lysates were immunoblotted (IB) for CD3ζ, p-Tyr and Lck. Lck phosphorylation were analyzed by immunoblotting for anti-pY394-Lck (anti-pY416-Src) or Lck of Lck IP products. Data are representative of three or more experiments with similar results.

Engagement of the TCR results in the activation of the *Src* family phosphotyrosine kinase (PTK), Lck[19], [20]. Activated Lck phosphorylates tyrosine residues in the ITAMs in the CD3ζ subunits of the TCR. ZAP-70 is then recruited to the phosphorylated CD3ζ and is activated by Lck[19], [21]. We previously reported that *Rhoh* ^-/-^ T cells demonstrate defective CD3ζ phosphorylation and ZAP-70 recruitment to the TCR complex which results in the impaired TCR signaling and T cell development[3]. We investigated whether impaired Lck activation could be responsible for this TCR signaling defect in RhoH-deficient thymocytes as well as peripheral T cells. While Lck *in vitro* kinase activity was similar in wild type (WT) and *Rhoh* ^-/-^ thymocytes (Fig. 1A), the association between Lck and CD3ζ and the phosphorylation of CD3ζ were impaired in *Rhoh* ^-/-^ T cells (Fig. 1A & B). Lck activity is regulated in a positive fashion by phosphorylation at tyrosine 394 (Y394)[22]. Consistent with the *in vitro* kinase activities noted above, the level of pY394 as demonstrated by immunoblot was comparable between WT and RhoH-deficient LN T cells (Fig. 1B). CD3ζ expression level was lower in *Rhoh* ^-/-^ thymocytes than WT (Fig. 1) due to the defective T cell development in *Rhoh* ^-/-^ mice with most *Rhoh* ^-/-^ thymocytes showing immature phenotype[3]. Published data has shown that CD3ζ expression is higher in single-positive thymocytes and peripheral T cells than in immature thymocytes[23]. These data suggest that while TCR signaling is abnormal, activation of Lck *per se* is not affected by loss of RhoH.

### RhoH requirement for co-recruitment of Lck and ZAP-70 to the immunologic synapse

**Figure 2:**
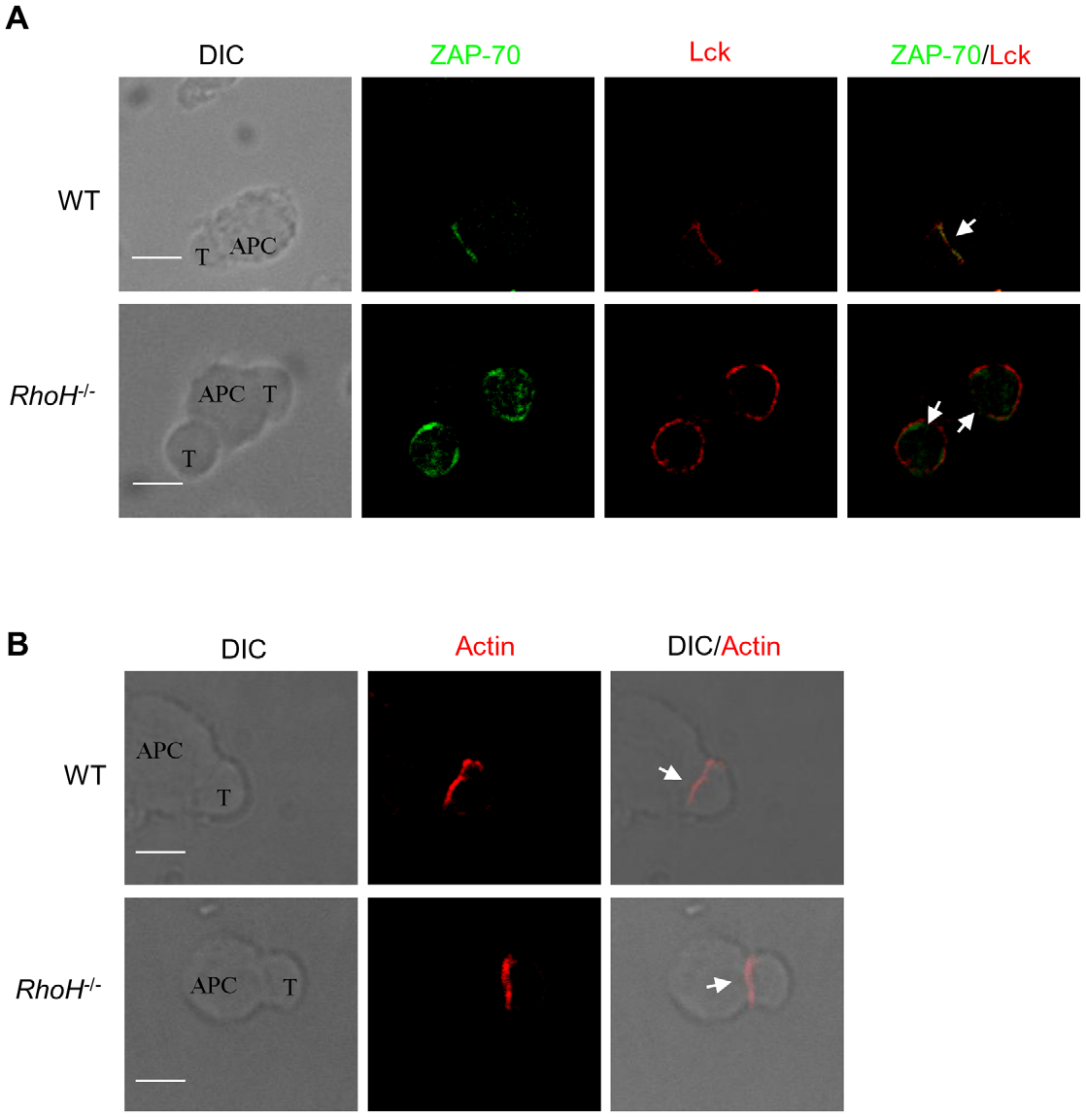
Impaired recruitment of Lck to the immunological synapse in *Rhoh^-/-^* T cells. (A) CD8^+^ T cells from WT or *Rhoh^-/-^* p14 TCR transgenic mice were conjugated with gp33 peptide-preloaded APC cells (CH.B2 cells) for 5 min. Cells were fixed and stained with anti-ZAP-70 (green) and anti-Lck (red). The localization of the immune synapse is indicated with a white arrow. Bars, 3 µm. (B) T cell-APC conjugates were fixed and stained with TRITC-Phalloidin for detection of F-actin. Differential interference contrast (DIC) images show the antigen-specific T cell-APC conjugates. Bars, 3 µm. At least 100 cell conjugates were examined per condition.

The interaction of T cells with antigen-presenting cells (APCs) results in the formation of the immunological synapse (IS). ZAP-70 has been reported to promote CD3ζ phosphorylation by recruiting Lck to TCR complex in a kinase activity-independent manner[9]. Both Lck and ZAP-70 have been shown to translocate to microclusters of receptors and signaling molecules in the peripheral regions of the T-APC interface during initiation of the TCR activation signal. The clusters then form a central supramolecular activation cluster (c-SMAC) of the IS[24], [25], [26]. Consistent with these previous observations, after conjugation with APCs we found in WT CD8^+^ cells that Lck translocated to the IS where it co-localized with ZAP-70 (Fig. 2A). In contrast, recruitment of both Lck and ZAP-70 to the IS was defective in *Rhoh* ^-/-^ T cells upon APC conjugation (Fig. 2A). This defect in IS localization was still present for up to 30 minutes after conjugation. Actin polymerization is critical for the formation of the IS by modulating the movement of molecules and lipid rafts[27], [28], and is important for integrin-dependent adhesion between T cell and APC[27]. Because Rho GTPases are known to be major regulators of actin polymerization, cytoskeleton assembly and integrin function, we examined the effect of RhoH-deficiency on actin polymerization in the IS. Actin polymerization was normal at the T-APC contact site in RhoH-deficient CD8^+^ T cells (Fig. 2B), while other markers such as CD8 and the TCRβ chain localize to the IS (data not shown). These data suggest that the defect in Lck and ZAP-70 translocation in RhoH-deficient T cells is not due to secondary effects on actin polymerization.

### Subcellular localization of Lck, CD3ζ, ZAP-70 and RhoH

**Figure 3:**
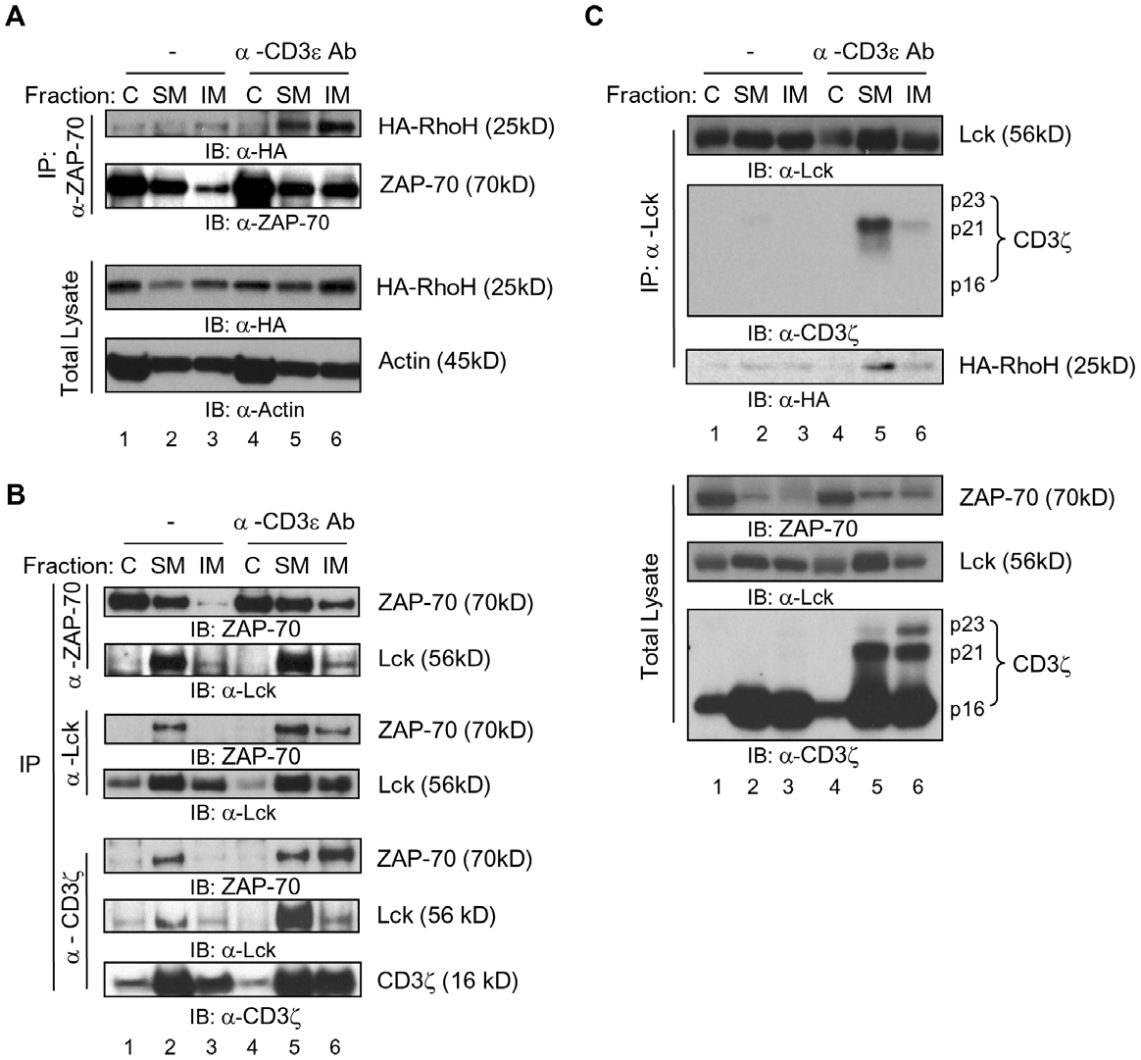
Lck, ZAP-70, CD3ζ and RhoH form multi-protein complexes in different subcellular fractions. Jurkat T cells transduced with a retroviral vector expressing HA-RhoH were stimulated or not with anti-CD3ε antibody before subcellular fractionation into cytosol (C), detergent-soluble (SM) and detergent-insoluble (IM) membrane fractions. Equal volumes of each fraction were immunoprecipitated using anti-ZAP-70, anti-Lck or anti-CD3ζ antibodies. (A) Interaction of RhoH with ZAP-70 in different subcellular fractions. IP products and total cell lysates as loading controls were immunoblotted for HA-RhoH (anti-HA antibody), ZAP-70 and β-actin as a loading control. (B) Interaction of ZAP-70, CD3ζ and Lck in different subcellular fractions. IP products were analyzed by immunoblotting for ZAP-70, Lck and CD3ζ. (C) Association of Lck with phospho-CD3ζ and RhoH in different subcellular fractions. IP products and total lysates were analyzed by immunoblotting for Lck, CD3ζ and HA-RhoH (anti-HA antibody). Figure shows data from one of at least three independent experiments.

Engagement of the TCR induces the translocation of several molecules such as Lck, ZAP-70 and CD3ζ to the detergent-insoluble membrane fraction of T cells that contains lipid rafts[29], [30], [31]. For further biochemical studies of the role of RhoH in Lck/ZAP-70/CD3ζ complex subcellular translocation, we expressed HA-tagged RhoH in Jurkat T cells. As measured by immunoblot, anti-CD3ε antibody stimulation led to the translocation of both HA-RhoH and ZAP-70 to the detergent-insoluble membrane fraction (31.47±2.7% to 46.34±4.08%, RhoH, unstimulated vs stimulated p<0.01; 11.93±2.29% to 24±4.92%, ZAP-70, unstimulated vs stimulated p<0.02; mean±SD; N = 3) with a significant amount of ZAP-70-bound HA-RhoH (Fig. 3A).

Since ZAP-70 is defective in translocation to the TCR complex and the detergent-insoluble membrane fraction in RhoH-deficient T cells[3], we next analyzed the association of ZAP-70 and Lck in different subcellular fractions by immunoprecipitation experiments. ZAP-70 co-immunoprecipitated with Lck in the detergent-soluble membrane fraction of transfected Jurkat T cells without stimulation. Treatment with anti-CD3ε Ab enhanced the association of ZAP-70 with Lck in the detergent-soluble and detergent-insoluble membrane fraction (upper panel in Fig. 3B). As expected from previous studies most of the complex remained in the detergent-soluble membrane fraction. Since Lck phosphorylates CD3ζ and phospho-CD3ζ recruits ZAP-70 to TCR complex to activate downstream signaling molecules[32], [33] we investigated the possible interactions of Lck with RhoH and CD3ζ. After anti-CD3ε stimulation most CD3ζ-bound ZAP-70 was detected in the detergent-insoluble membrane (Fig. 3 B, lower panel). In these studies, fully phosphorylated CD3ζ (p23) localized mostly to the detergent-insoluble membrane (Fig. 3C, lower panel) and Lck co-immunoprecipitated primarily with the phosphorylated form of CD3ζ in anti-CD3ε stimulated cells. RhoH together with CD3ζ was associated with Lck and ZAP-70 as determined by co-immunoprecipitation analysis after anti-CD3ε stimulation. Lck also co-immunoprecipitated with HA-RhoH in the detergent-soluble membrane fraction after anti-CD3ε Ab stimulation (Fig. 3B & C).

### Enhancement of ZAP-70/RhoH complex formation by Lck

**Figure 4:**
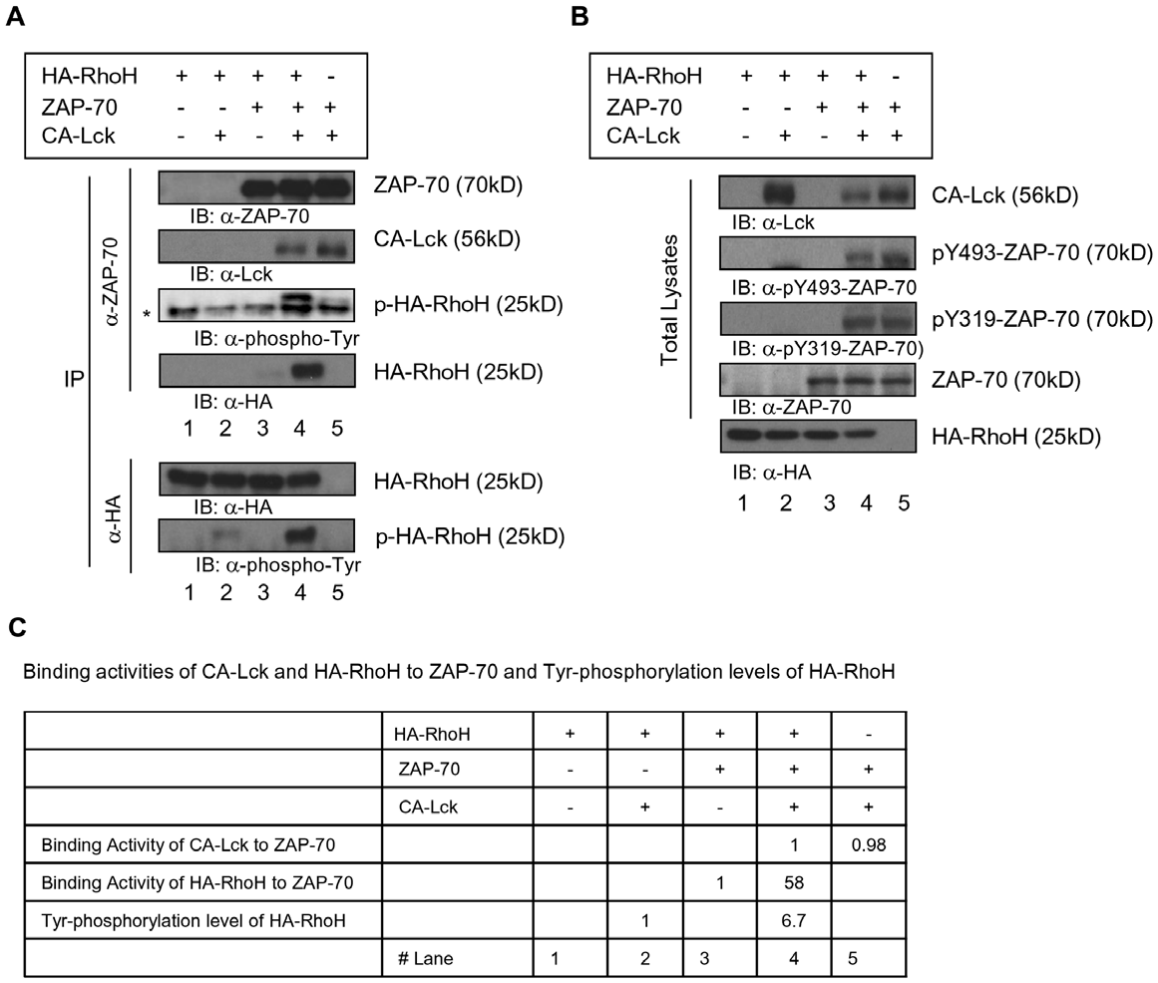
Lck enhances the association of RhoH with ZAP-70. HEK293 cells were transfected with combinations of plasmids expressing HA-RhoH, constitutively active Lck (CA-Lck), or ZAP-70. Total lysates of transfected HEK293 cells were immunoprecipitated using anti-HA or anti-ZAP-70 antibodies. IP products (A) and total lysates (B) were analyzed by immunoblotting. Asterisks denote IgG light chains. (C) Binding activities of CA-Lck and HA-RhoH to ZAP-70 and Tyr-phosphorylation levels of HA-RhoH. Relative amounts of bound ZAP-70 or total protein levels of the Lck and HA-RhoH and Tyr-phosphorylated HA-RhoH in (A) and (B) were quantified by densitometric measurements. Binding activities were calculated by the ratio of protein amounts of CA-Lck and HA-RhoH bound in ZAP-70 IP products to total lysates. Figure shows data from one of at least three independent experiments.

Binding of ZAP-70 to the ITAM motifs of CD3ζ has been suggested to induce a conformational change of ZAP-70 that facilitates the phosphorylation of Tyr315 and Tyr319 in interdomain B of ZAP-70[17], [34]. These phosphorylation events establish the active conformation of ZAP70. We have previously shown that RhoH associates with ZAP-70 in a phosphorylation-dependent manner[3]. To further study the potential formation of multi-protein complexes containing RhoH, we transfected HEK293 cells with RhoH, ZAP-70 and a constitutive active mutant of Lck (CA-Lck). Exogenously-expressed RhoH did not affect the association of ZAP-70 and Lck (Fig. 4A, upper panel, lane 4 vs lane 5 and summarized in C) or the phosphorylation on Tyr319 and Tyr493 of ZAP-70 by CA-Lck (Fig. 4B, lane 4 vs lane 5). CA-Lck alone did not phosphorylate RhoH to the same level as co-expression of CA-Lck and ZAP-70(Fig. 4A, lower panel, lane 2 vs lane 4 and C). Expression of CA-Lck, ZAP-70 and RhoH together enhanced the interaction of ZAP-70 with RhoH and the phosphorylation of RhoH (Fig. 4A, upper panel, lane 4 and lower panel and C). These data and the fact that RhoH did not co-immunoprecipitate with Lck in the absence of ZAP-70 suggest that Lck does not interact directly with RhoH in the absence of ZAP-70.

**Figure 5:**
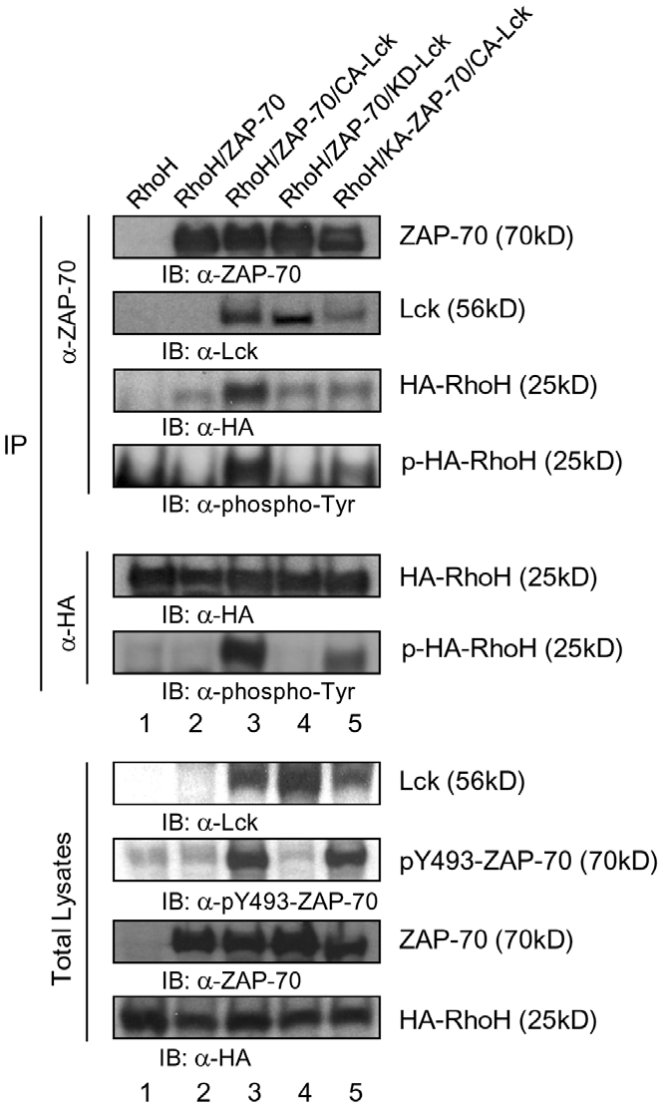
Both ZAP-70 and Lck kinase activities are required for the association of RhoH with ZAP-70. HEK293 cells were transfected with combinations of the plasmids expressing HA-RhoH, CA-Lck, kinase-dead Lck (KD-Lck), ZAP-70 or kinase-dead ZAP-70 (KD-ZAP-70). Total lysates were submitted to immunoprecipitation (IP) of anti-ZAP-70 or anti-HA Ab and immunoprecipitates and total lysates were analyzed by immunoblotting.

Since CA-Lck alone did not phosphorylate RhoH efficiently, we next investigated whether ZAP-70 could phosphorylate RhoH with or without expression of Lck. As noted above, co-expression of CA-Lck, ZAP-70 and RhoH led to increased phosphorylation of RhoH which is immunoprecipitated with ZAP-70 (Fig. 5, lane 3). A co-expressed kinase-dead mutant of Lck (KD-Lck) and ZAP-70 did not complex with RhoH and was not associated with phosphorylation of RhoH (Fig. 5, lane 4). While co-expression of a kinase-dead ZAP-70 mutant (KA-ZAP-70) with CA-Lck could still lead to increased phosphorylation of RhoH, CA-Lck did not enhance the association of ZAP-70 with RhoH and KA-ZAP-70 also showed lower affinity to CA-Lck. (Fig. 5, lane 5). These data suggest that the kinase activities of both ZAP-70 and Lck are required for the association of RhoH with ZAP-70 and efficient phosphorylation of RhoH.

**Figure 6:**
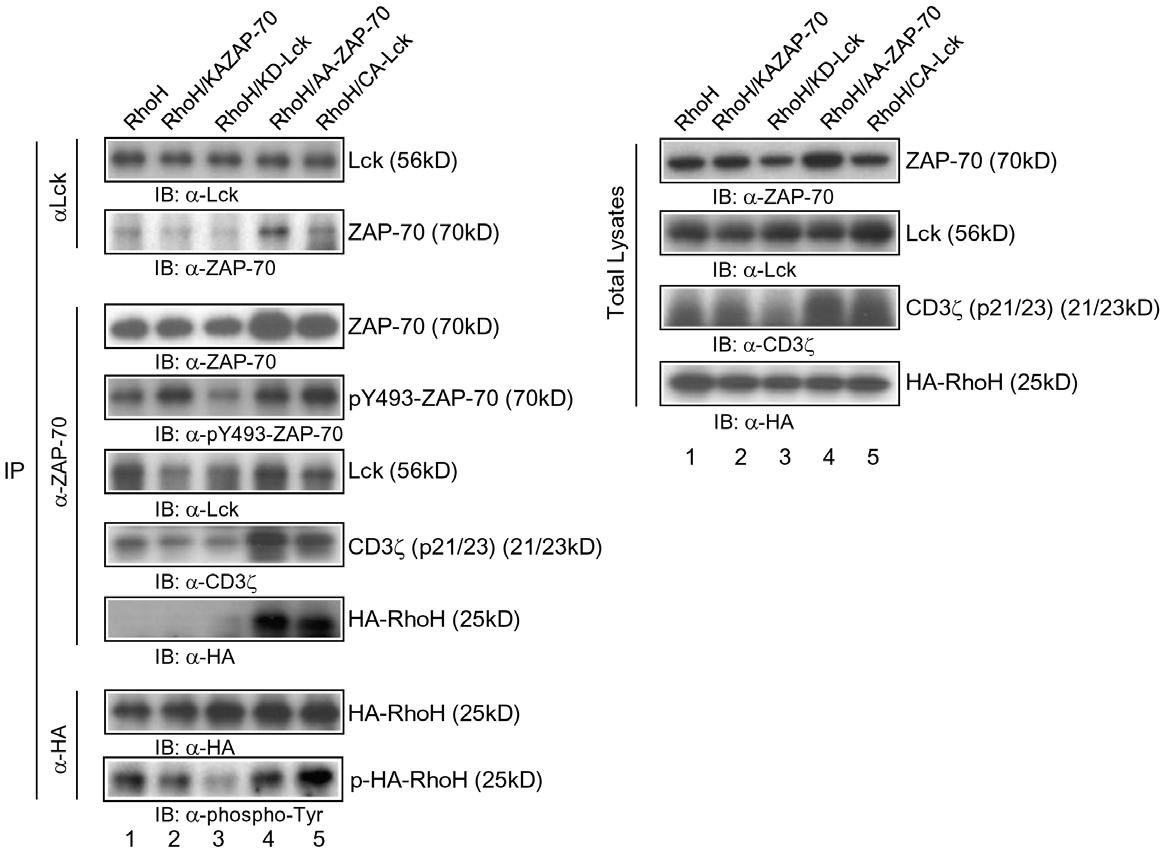
Constitutive active Lck enhances the phosphorylation and ZAP-70 association of RhoH in Jurkat T cells. Jurkat T cells transduced with a retroviral vector expressing HA-RhoH were transfected with combinations of the plasmids expressing EGFP, CA-Lck, kinase-dead Lck (KD-Lck), constitutive active ZAP-70 (AA-ZAP-70) or kinase-dead ZAP-70 (KA-ZAP-70). Total lysates were submitted to immunoprecipitation (IP) of anti-ZAP-70, anti-Lck or anti-HA Ab and immunoprecipitates and total lysates were analyzed by immunoblotting.

We next investigated whether Lck and ZAP-70 activity is required for the association of ZAP-70 with RhoH and phosphorylation of RhoH in T cells (Fig. 6). Expression of CA-Lck in Jurkat T cells induced the phosphorylation of ZAP-70, CD3ζ, and RhoH. CA-Lck also enhanced the association of ZAP-70 with RhoH (Fig. 6, lane 5 vs. lane 1). In contrast, KD-Lck inhibited the phosphorylation of CD3ζ, ZAP-70, and RhoH (Fig. 6, lane 3) and reduced the association of RhoH with ZAP70 (Fig.6, lane 3 vs lane 6). Jurkat T cells expressing a constitutive active ZAP-70 mutant (AA-ZAP-70) showed enhanced association between RhoH and ZAP-70, but not phosphorylation of RhoH (Fig. 6, lane 4 vs lane 1). AA-ZAP-70 induced the CD3ζ phosphorylation and ZAP-70/Lck complex formation. (KA-ZAP-70) did not alter the phosphorylation of RhoH and CD3ζ (Fig. 6). As noted in HEK293 cells, both CA-Lck and AA-ZAP-70 enhanced the co-association of ZAP-70/RhoH/CD3ζ complex formation, but KA-ZAP-70 inhibited ZAP-70/Lck association in Jurkat T cells as well as HEK293 cells (Fig. 5 & 6). These data suggest that Lck phosphorylates RhoH and CD3ζ through the interaction with functional ZAP-70.

### Myristoylation sequence tagged ZAP-70 can partially rescue the defective TCR signaling and thymic development in *Rhoh^-/-^* thymocytes

Transient or stable membrane association of ZAP-70 mediated by the membrane-permeable synthetic ligand FK1012A or by a myristoylation sequence -tagged ZAP70 (Myr-ZAP-70) can activate downstream signaling of TCR[35], [36]. Since ZAP-70 does not translocate to the TCR complex in Rho-deficient T cells, to determine if defective T cell development in *Rhoh* ^-/-^ mice was due to defective ZAP-70 membrane localization, we generated a retroviral vector expressing Myr-ZAP-70 or control vector and utilized these vectors to transduce *Rhoh-/-* low density bone marrow cells (LDBM). First to determine the functional consequences of Myr-ZAP-70, we transduced Ba/F3 cells. Expression of the Myr-ZAP-70 in Ba/F3 cells led to an increase in the proportion of ZAP-70 protein in the detergent-soluble (27% to 40%) and detergent-insoluble (6% to 22%) membrane fractions of these cells (Fig. S1A). We subsequently compared the subcellular localization of Myr-ZAP-70 with WT-ZAP-70 in the transduced LDBM cells. Most Myr-ZAP-70 was localized to the plasma membrane in these transduced cells (Fig. S1B).

**Figure 7:**
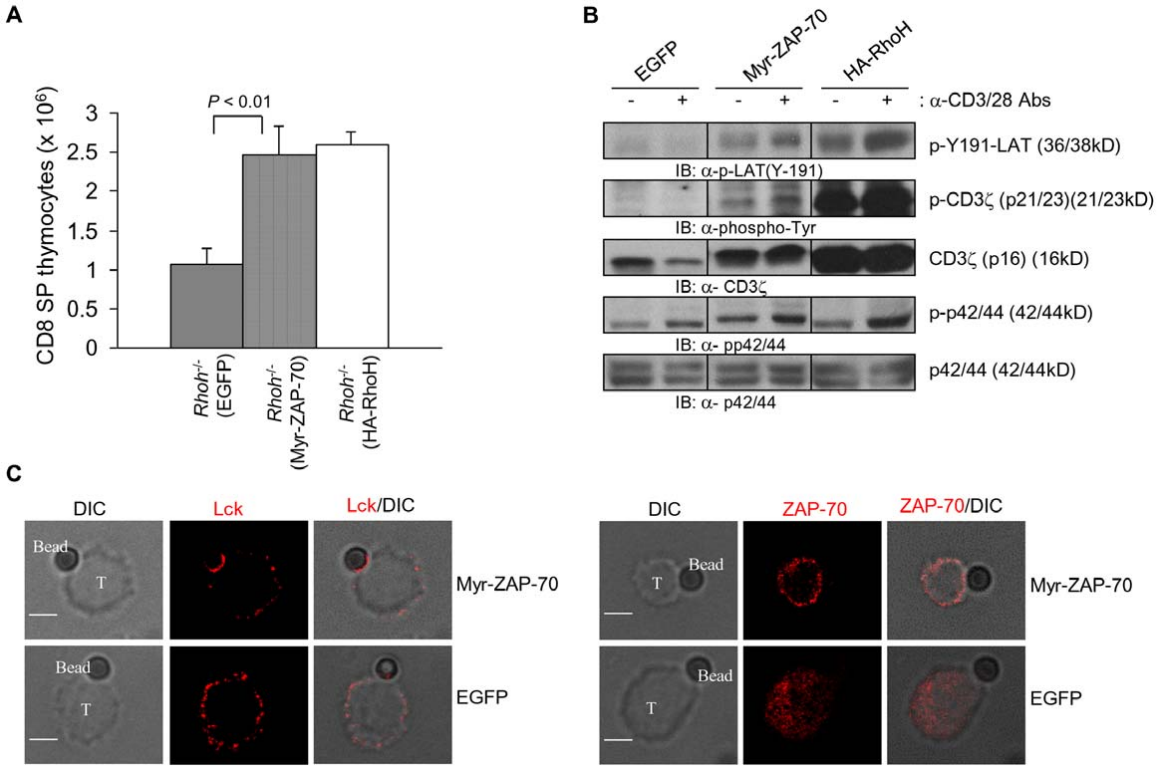
Membrane targeting of ZAP-70 partially rescues defective *Rhoh* ^-/-^ thymic development. *Rhoh* ^-/-^ LDBM cells were transduced with a retroviral vector co-expressing Myr-ZAP-70, HA-RhoH and EGFP or EGFP alone as a control. EGFP^+^-sorted cells were injected intravenously into sub-lethally irradiated *Rag2* ^-/-^ recipient mice. Thymocytes from *Rag2* ^-/-^ recipient mice were analyzed 8 weeks after transplantation by flow cytometry (A) and immunoblotting (B). (A) Number of CD8 single positive cells in the thymus of *Rag2* ^-/-^ recipient mice. Thymocytes from the recipient mice were analyzed for CD4/CD8 subsets by flow cytometry. CD8 SP thymocytes were calculated by (frequency) x (cellularity). Data represent the mean +/- SD. N = 3 mice per transplant group. (B) Phosphorylation of CD3ζ, LAT and p44/42 in Myr-ZAP-70-expressing *Rhoh* ^-/-^ thymocytes. Thymocytes from *Rag2* ^-/-^ recipient mice were left unstimulated or stimulated anti-CD3ε and CD28 mAbs for 2 min. Total lysates were separated on a SDS-polyacrylamide gel then transferred to PVDF membranes and immunoblotted for CD3ζ, p-Tyr, p-p44/42, p44/42 and p-LAT. (C) Subcellular localization of Lck in *Rhoh* ^-/-^ T cells expressing Myr-ZAP-70. *Rhoh* ^-/-^ T cells were transduced with retroviruses co-expressing Myr-ZAP-70 and EGFP or EGFP alone. EGFP^+^ T cells were incubated with biotin-labeled anti-CD3 and CD28 Abs, and then conjugated with Dynabeads Biotin Binder for 5 min. Cells were fixed and stained with anti-ZAP-70 or anti-Lck Ab. DIC images show the T cell-microbead conjugates. Bars, 3 µm. At least 100 cell conjugates were examined per condition.

Next, we analyzed the engraftment and T cell development of Myr-ZAP-70-expressing *Rhoh^-/-^* bone marrow cells transplanted into *Rag2^-/-^* mice. *Rhoh* ^-/-^ bone marrow (BM) cells were transduced with a bi-cistronic retroviral vector co-expressing enhanced green fluorescent protein (EGFP) and HA-RhoH, Myr-ZAP-70 or EGFP alone (as control). Transduced and EGFP^+^-sorted BM cells were transplanted via intravenous injection into the sub-lethally irradiated Rag2-deficient recipient mice. Development of CD8 single positive (SP) thymocytes at 8 weeks post-transplantation in *Rag2^-/-^* mice infused with Myr-ZAP-70-transduced *RhoH* ^-/-^ BM cells was significantly increased compared with mice transplanted with *Rhoh* ^-/-^ BM cells transduced with the empty-vector and reached levels equivalent to mice transplanted with cells transduced and expressing the RhoH cDNA (Fig. 7A). In addition, there was a trend toward correction of CD4 SP thymocytes in these mice, although this correction did not reach statistical significance (2.39±0.88 *vs* 16.75±14.25×10^6^; CD4 SP, mean ± SD; EGFP *vs* Myr-ZAP-70; N = 3). Total thymic cellularity was also increased in mice transplanted with Myr-ZAP-70-transduced *Rhoh* ^-/-^ BM cells (44.6±3.7×10^6^/mouse vs 104.8±45.6×10^6^; mean ± SD; EGFP vs Myr-ZAP-70; N = 3; p<0.05).

We next investigated whether TCR signaling was corrected in *Rhoh^-/-^* thymocytes expressing Myr-ZAP-70. As previously noted[3], we confirmed that *Rhoh^-/-^* thymocytes showed impaired activation of LAT, CD3ζ and p44/42 (Fig. 7), all molecules involved in TCR signaling. Expression of Myr-ZAP-70 partially restored the activation of these molecules after anti-CD3/28 Abs stimulation. While we could not detect the phosphorylation of CD3ζ and LAT after anti-CD3/28 Abs stimulation in *Rhoh^-/-^* thymocytes expressing EGFP alone, expession of Myr-ZAP-70 in *Rhoh^-/-^* thymocytes led to increased phosphorylation of CD3ζ and LAT (Fig. 7B). To examine whether the defective translocation of Lck to the immunologic synapse was corrected in *Rhoh^-/-^* T cells expressing Myr-ZAP-70, we used anti-CD3/CD28 conjugated microbeads to activate T cells and induce IS formation[37], [38]. We transduced *Rhoh-/-* lymph node (LN) T cells with retroviruses co-expressing EGFP and Myr-ZAP-70 or EGFP alone. EGFP^+^ sorted LN T cells were incubated with anti-CD3ε and CD28 Abs, and then conjugated with Dynabeads. Lck was polarized towards the microbead interface of *Rhoh^-/-^* T cells expressing Myr-ZAP-70, while Lck was widely dispersed over the membrane of *Rhoh^-/-^* T cells expressing only EGFP (Fig. 7C left panel). The association of ZAP-70 with the T cell membrane was enhanced by expression of Myr-ZAP-70 compared with T cells expressing EGFP alone (Fig. 7C right panel). These data demonstrate that RhoH functions as an adaptor for ZAP-70 to facilitate the translocation of Lck into the TCR complex and membrane-targeted ZAP-70 can partially correct the defective TCR signaling in *Rhoh^-/-^* T cells.

## Discussion

RhoH is an hematopoietic-specific GTPase-deficient GTPase of the RhoE family first identified as a fusion of LAZ3/BCL6 in non Hodgkin's Lymphoma (NHL)[1]. Subsequently, RhoH has been found to be mutated in multiple myeloma and diffuse large B cell lymphomas (DLBCL)[39], [40] and in AIDS-associated NHL[41], although the pathophysiological relevance of these findings are still unknown. Due to the presence of alternative residues at the highly conserved amino acids analogous to positions 12 and 61 of the Ras proteins regulating GTPase activity, RhoH remains GTP bound. Thus, the cellular activity and function of RhoH has been hypothesized to be dependent on protein levels in the cell.

Previous studies have implicated RhoH in T cell development and TCR signaling, inside-out integrin signaling and adhesion in T cells and antagonism of the activity of the Rac GTPase pathways in cells of hematopoietic lineages[4], [5], [7], [42]. Using gene-targeted mice deficient in RhoH, we have previously demonstrated defective thymocyte development, positive and negative selection and TCR signaling. *Rhoh^-/-^* mice have T lymphopenia and demonstrate reduced TCR-induced T cell proliferation *in vitro*. Proteomic studies demonstrated that RhoH interacts with ZAP-70 in TCR signaling, providing an initial insight into the molecular mechanism of defective T cell signaling in *Rhoh^-/-^* cells.

Here we show that RhoH plays a critical role in facilitating the localization of multiple TCR signaling components-likely via the ZAP-70 interaction-to the membrane, particularly the detergent insoluble fraction of the membrane that contains lipid rafts. Lck and ZAP-70 have been shown to translocate to microclusters of receptors and signaling molecules in the peripheral regions of the T-APC interface within lipid rafts during initiation of the TCR activation signal. The clusters form a central supramolecular activation cluster (c-SMAC) of the IS [24], [25], [26]. In addition, ZAP-70 has been reported to promote CD3ζ phosphorylation by recruiting Lck to TCR complex in a kinase activity-independent manner[9]. In this regard, although Lck kinase activity appears normal in the absence of RhoH, here we demonstrate the *Rhoh^-/-^* T cells show defective ZAP-70 and Lck localization to the IS and defective downstream signaling of the TCR pathway. Our studies demonstrate protein interactions between Lck, ZAP-70 and RhoH and that the translocation of both ZAP-70 and Lck to the IS is decreased in *Rhoh^-/-^* T cells. Thus, RhoH appears to be involved in the coordinated movement of proteins in the IS. We are currently studying which domains in addition to the ITAM-like motifs of RhoH are critical for these interactions.

Previous studies have implicated the translocation of Lck, ZAP-70 and the TCR into the IS in effective TCR signaling [29], [30], [31]. Binding of ZAP-70 to ITAM motifs of CD3ζ has been suggested to induce a conformational change of ZAP-70 to facilitate the phosphorylation of Tyr315 and Tyr319 in interdomain B of ZAP-70, which establishes and stabilizes the active conformation of ZAP-70[17], [34]. We have previously shown that RhoH associates with ZAP-70 in phosphorylation-dependent manner[3]. In the studies reported here, exogenously-expressed RhoH alone did not affect the association of ZAP-70 and Lck or the phosphorylation of ZAP-70 on Tyr319 and 493 of ZAP-70 by Lck. However, expression of CA-Lck and functional ZAP-70 together enhanced the interaction of ZAP-70 with RhoH and the phosphorylation of RhoH. The motifs involved in these interactions are currently under additional investigations. RhoH-dependent localization of Lck to the IS via ZAP-70 would suggest that ZAP-70 can function both upstream and downstream of Lck in the localization of TCR signaling components and phosphorylation of CD3ζ. This is consistent with previous reports that TCR-induced phosphorylation of CD3ζ is reduced in *ZAP-70^-/-^* mice, suggesting a role for ZAP-70 as an upstream facilitator of Lck localization and function[43].

Taken together, these data suggest that RhoH may act as an adaptor molecule for ZAP-70 and Lck recruitment which, in turn, phosphorylates RhoH to enhance the heterotrimeric association of these molecules. In support of this hypothesis, CA-Lck is required for the activation of ZAP-70 and co-expression of CA-Lck and ZAP-70 enhanced the phosphorylation and binding affinity of RhoH to ZAP-70. Since CA-Lck alone did not phosphorylate RhoH efficiently, we also investigated the potential role of ZAP-70 in RhoH phosphorylation. Co-expression of kinase-dead Lck and ZAP-70 did not result in measurable phosphorylation of RhoH. However, a constitutive active mutant of Lck induced phosphorylation of RhoH and enhanced interaction among ZAP-70/Lck/RhoH in Jurkat T cells, whereas kinase-dead Lck inhibited the phosphorylation of RhoH and CD3ζ. Constitutive active ZAP-70 also enhanced ZAP-70/RhoH complex formation through Lck activation because AA-ZAP-70 showed enhanced CD3ζ phosphorylation and association with Lck in Jurkat T cells. Co-expression of kinase-dead ZAP-70 with CA-Lck resulted in reduced phosphorylation of RhoH, and the KA-ZAP-70 did not co-immunoprecipitate with RhoH and showed lower binding affinity to Lck. These data suggest that functional ZAP-70 is required for the Lck-mediated RhoH phosphorylation and optimal association of RhoH with ZAP-70.

Supporting a critical role for RhoH in functionally important localization of ZAP-70 to the membrane, anti-CD3/28 Abs-induced phosphorylation of CD3ζ and LAT was partially restored in *Rhoh^-/-^* thymocytes expressing Myr-ZAP-70. As expected, a higher proportion of Myr-ZAP-70 compared with wild-type ZAP-70 showed association with the cell membrane in T cells. Myr-ZAP-70 partially rescued in thymocyte development and TCR signaling in the absence of RhoH. Membrane-bound ZAP-70 can activate TCR minimally without stimuli, but still requires functional Lck activity [35], [36]. Since Myr-ZAP-70 can partially rescue the thymocyte development and TCR signaling in *Rhoh^-/-^* cells, we suggest that RhoH function is required for the membrane localization of ZAP-70, particularly to the IS. In the experiments reported here, there could be two reasons why Myr-ZAP-70 did not completely restore the TCR signaling in *Rhoh^-/-^* cells completely. First, the sustained TCR activation by membrane-bound ZAP-70 might induce a refractory state that reduces TCR signaling efficiency as previously reported[35]. Second, src myristoylation sequence broadly localizes ZAP-70 to the membrane, and not to specific TCR signaling regions (Fig. S1)[35]. In *Rhoh^-/-^* T cells expressing Myr-ZAP-70, Lck was polarized towards the interface of cells and microbeads coated with anti-CD3ε and anti-CD28 antibodies, while Lck remained dispersed over the entire membrane in *Rhoh^-/-^* T cells expressing only EGFP. These data strongly suggest that RhoH functions with ZAP-70 to also facilitate the translocation of Lck into the TCR complex. Taken together with the data showing partial rescue of T cell development after *Rhoh-/-* cells expressing Myr-ZAP-70 are transferred into *Rag2-/-* mice, these data suggest that this function of RhoH is physiologically important.

Other investigators have confirmed that RhoH promotes the ZAP70-dependent phosphorylation of the LAT signalosome[7]. Although they did not show defective localization and activation of ZAP-70, there was complete loss of CD3ζ phosphorylation[44]. One possibility to explain the difference in ZAP-70 phosphorylation in these studies is that this group stimulated T cells using CD3 and CD4 co-crosslinking, which could lead to the direct recruitment of Lck.

Altogether, these data suggest that Lck/ZAP-70 complex may facilitate the phosphorylation of RhoH, which then enhances the interaction between ZAP-70 and RhoH. The enhanced association between RhoH and ZAP-70 may then facilitate the localization of Lck/ZAP-70 to the TCR complex leading to the known role of these proteins in phosphorylation of CD3ζ. After this, ZAP-70 bound to CD3ζ, ZAP-70/CD3ζ or ZAP-70/RhoH moves to the detergent-insoluble fraction or IS region. Overall, while RhoH appears to regulate Rac activity in some hematopoietic cell lines and in primary hematopoietic cells, the data presented here and the differences in T cell phenotypes of *Rhoh^-/-^, Rac1^-/-^;Rac2^-/-^, ZAP-70^-/-^* and *Vav1^-/-^* (reviewed in Wang and Zheng[45]) mice suggest that RhoH functioning in TCR signaling and T cell development is more complicated and in part related to its function as an adaptor molecule that affects localization of both ZAP-70 and Lck in the IS. This defines a novel function of Rho GTPases.

## Supporting Information

Figure S1 Membrane localization of Myr-ZAP-70. (A) Lysates of Ba/F3 cells transduced with retroviral vectors expressing ZAP-70 or Myristoylated ZAP-70 (Myr-ZAP-70) were separated by centrifugation into cytosol (C), detergent-soluble (SM) and detergent-insoluble (IM) membrane fractions. Equal volumes of each fractions were immunoblotted for ZAP-70 and β-actin as a loading control. (B) LDBM cells from wild type mice were infected with retroviral vectors expressing ZAP-70, Myr-ZAP-70 or EGFP alone. The transduced cells were fixed and stained with anti-ZAP-70 (red). Bars, 3 µm.
(4.38 MB TIF)
