# Supplementary figures and images for: RhoH Regulates Subcellular Localization of ZAP-70 and Lck in T Cell Receptor Signaling
Source: PLoS One. 2010 Nov 12;5(11):e13970. doi: 10.1371/journal.pone.0013970 (PMC2980477; doi:10.1371/journal.pone.0013970)

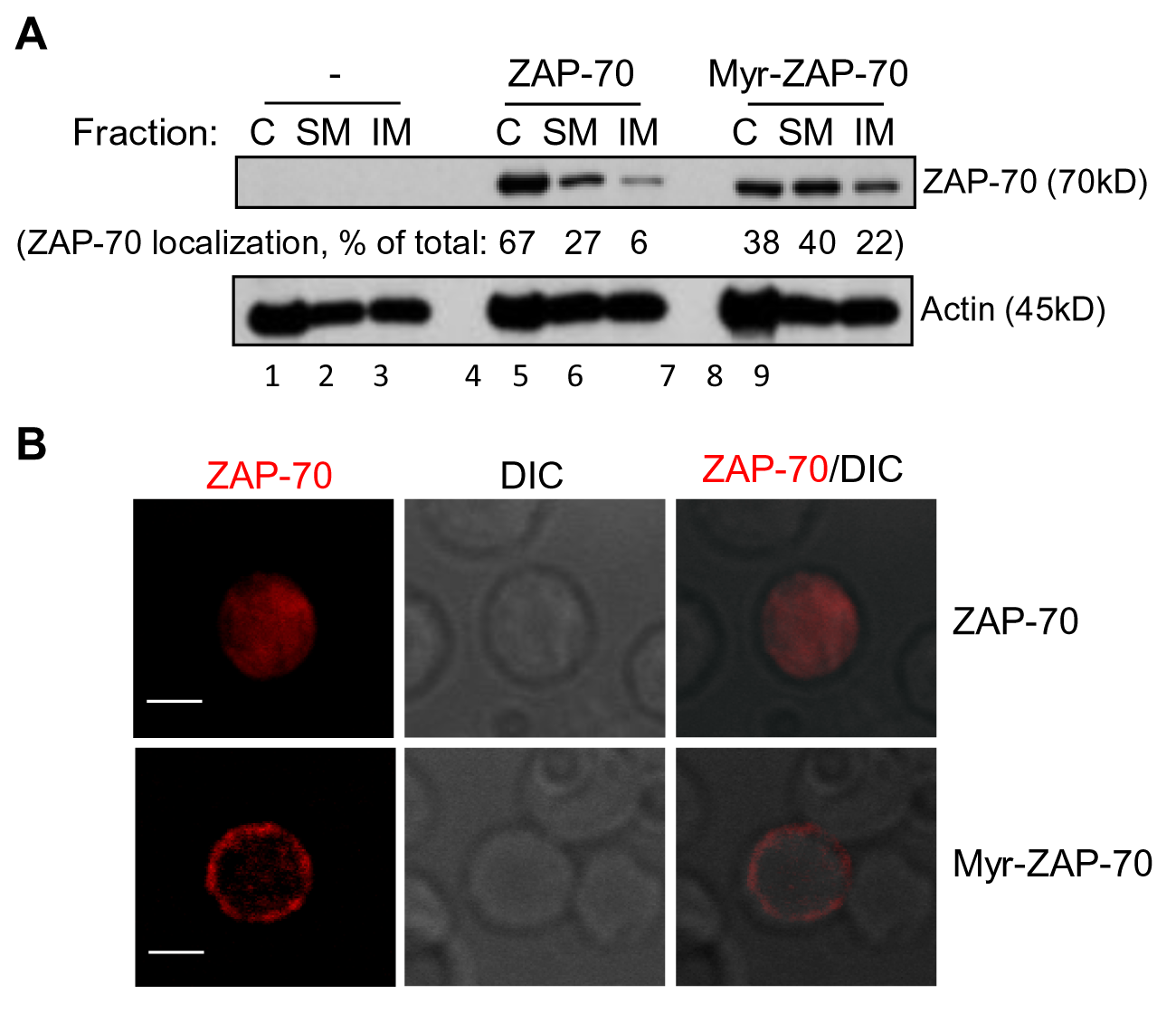

Supplement: Figure S1 — Membrane localization of Myr-ZAP-70. (A) Lysates of Ba/F3 cells transduced with retroviral vectors expressing ZAP-70 or Myristoylated ZAP-70 (Myr-ZAP-70) were separated by centrifugation into cytosol (C), detergent-soluble (SM) and detergent-insoluble (IM) membrane fractions. Equal volumes of each fractions were immunoblotted for ZAP-70 and β-actin as a loading control. (B) LDBM cells from wild type mice were infected with retroviral vectors expressing ZAP-70, Myr-ZAP-70 or EGFP alone. The transduced cells were fixed and stained with anti-ZAP-70 (red). Bars, 3 µm. (4.38 MB TIF) [file pone.0013970.s001.tif]
